# Associations of vitamin status and T-lymphocyte subsets with tic severity in children: a cross-sectional study using the Yale Global Tic Severity Scale

**DOI:** 10.3389/fpsyt.2026.1903047

**Published:** 2026-10-14

**Authors:** Fengming Zhu, Yun Gao, Zhenlong Zhang, Shumin Kang, Weidong Xu, Jian Zhang

**Affiliations:** Department of Pediatrics, Zhangjiagang Hospital Affiliated to Soochow University/The First People’s Hospital of Zhangjiagang City, Zhangjiagang, Jiangsu, China

**Keywords:** CD4+ T lymphocytes, pediatric neuroimmunology, tic disorder, Tourette syndrome, vitamin D, Yale Global Tic Severity Scale

## Abstract

**Background:**

Vitamin D insufficiency and immune dysregulation have been implicated in tic disorders; however, the relationship between vitamin status, peripheral T-lymphocyte subsets, and tic severity in children remains incompletely defined.

**Objective:**

We aimed to evaluate associations between serum vitamin indices, peripheral lymphocyte subsets, and tic severity, measured by the Yale Global Tic Severity Scale (YGTSS) global severity score, in newly diagnosed children with tic disorders.

**Methods:**

This single-center cross-sectional study included children aged <14 years with newly diagnosed, treatment-naive tic disorders who attended Zhangjiagang Hospital affiliated to Soochow University/The First People’s Hospital of Zhangjiagang City between January 2022 and August 2025. The YGTSS global severity score was calculated as the sum of the total tic score and impairment score. Normality was assessed using the Shapiro–Wilk test. Associations were examined using Spearman correlation with Benjamini–Hochberg FDR correction and adjusted linear regression with HC3 robust standard errors. Vitamin D groups were compared using the Kruskal–Wallis test followed by Dunn–Bonferroni pairwise tests. Analyses were performed using IBM SPSS Statistics version 27.0 and R version 4.4.2.

**Results:**

In total, 41 children were included (29 male and 12 female participants; mean age, 8.61 ± 2.70 years). The mean YGTSS global severity score was 30.71 ± 10.25. CD4^+^ T-cell absolute count showed the strongest inverse correlation with the YGTSS global severity score (Spearman *ρ* = −0.474, *P* = 0.0017; FDR-adjusted *P* = 0.0260). After adjustment for age, sex, and disease duration, the association remained significant using HC3 robust standard errors (*B* = −0.018, 95% CI −0.031 to −0.005; *P* = 0.0069). The overall difference among vitamin D groups was significant (Kruskal–Wallis *P* = 0.0073); Dunn–Bonferroni testing identified a difference only between the <20- and 20- to <30-ng/mL groups (adjusted *P* = 0.0077).

**Conclusion:**

Lower peripheral CD4^+^ T-cell absolute count remained associated with greater tic-related symptom burden after covariate adjustment in children with tic disorders. Vitamin D findings were exploratory, did not show a monotonic dose–response pattern, and were less robust than the CD4^+^ T-cell association. Larger prospective studies are needed.

## Introduction

Tic disorders, including Tourette syndrome and chronic motor or vocal tic disorder, are childhood-onset neurodevelopmental conditions characterized by recurrent motor and/or vocal tics that fluctuate over time (1, 2). Accurate assessment of tic severity is essential for clinical phenotyping, monitoring disease burden, and evaluating treatment response. The Yale Global Tic Severity Scale (YGTSS) is the most widely used clinician-rated instrument for quantifying tic severity and has been supported by psychometric studies and guideline recommendations (3–7).

Although dysfunction of cortico-striato-thalamo-cortical circuits remains central to current models of tic disorders, increasing evidence suggests that immune and inflammatory mechanisms may contribute to tic expression or exacerbation in a subset of children (1, 8, 9). Altered cytokine profiles, lymphocyte subset abnormalities, and associations with allergic or post-infectious conditions have been reported in pediatric tic disorders and Tourette syndrome (10–14). These observations suggest that peripheral immune measures may provide useful, although still exploratory, information about tic-related disease activity.

Vitamin D is an immunomodulatory secosteroid that influences innate and adaptive immune responses, antigen-presenting cells, cytokine production, and T-cell differentiation (15–17). Previous studies have reported lower serum 25-hydroxyvitamin D [25(OH)D] levels in children with chronic tic disorders, and one small supplementation study suggested symptom improvement after vitamin D_3_ supplementation (18). A recent systematic review and meta-analysis also found lower vitamin D levels in children with tic disorders than in healthy controls, while emphasizing the limitations of existing observational evidence (19).

Other fat-soluble vitamins were included for exploratory biological reasons. Vitamin A-derived retinoic acid regulates T-cell differentiation, trafficking, and immune tolerance (20). Vitamin E is a membrane antioxidant that modulates innate and adaptive immune cell function (21). Vitamin K participates in sphingolipid metabolism and vitamin K-dependent signaling in neural tissues, although evidence in tic disorders is sparse (22). These mechanistic observations do not establish clinical associations with tics; they provide a rationale for exploratory screening of vitamins A, E, and K alongside vitamin D.

Few studies have simultaneously examined serum vitamin status and peripheral T-lymphocyte subsets in relation to YGTSS global severity scores. We therefore conducted an exploratory cross-sectional study to evaluate associations between the YGTSS global severity score, serum vitamin indices, and lymphocyte subset parameters in newly diagnosed, treatment-naive children with tic disorders. Vitamin D was the primary vitamin of interest, whereas vitamins A, E, and K were evaluated as exploratory nutritional markers. We hypothesized that lower vitamin D levels and immune subset alterations, particularly lower CD4^+^ T-cell absolute count, would be associated with greater tic-related symptom burden.

## Methods

### Study design and participants

This was a single-center cross-sectional study using consecutively collected clinical data from children who attended Zhangjiagang Hospital affiliated to Soochow University/The First People’s Hospital of Zhangjiagang City between January 2022 and August 2025. All included participants were newly diagnosed at their first clinical visit and had not received tic-specific pharmacological treatment before assessment. A total of 41 children with available YGTSS assessment, serum vitamin measurements, and lymphocyte subset testing were included.

The inclusion criteria were as follows: (1) fulfillment of the DSM-5 diagnostic criteria for tic disorders, (2) complete clinical data, (3) age <14 years, and (4) written informed consent from a parent or legal guardian with the child’s assent or cooperation as developmentally appropriate.

The exclusion criteria were as follows: (1) uncertain diagnosis or another documented genetic, neurological, or major psychiatric disorder; (2) clinically evident infectious disease or a recent infectious event documented in the clinical history, chronic disease, malignant disease, or a known immunological or autoimmune disorder; and (3) recent use of medications or vitamin supplements. Eligibility was determined from the clinical history and review of medical records. High-sensitivity C-reactive protein was not available as a standardized screening measure for all participants.

Demographic and clinical variables included age, sex, and disease duration. Diagnoses were established according to DSM-5 criteria at the first clinical visit. All included participants were newly diagnosed and treatment-naive.

The study was reviewed and approved by the Ethics Committee of Zhangjiagang Hospital affiliated to Soochow University/The First People’s Hospital of Zhangjiagang City (approval no. ZJGYYLL-2022-10-013). All participants and their parents or legal guardians were informed about the study, and written informed consent was obtained from the parents or legal guardians prior to participation.

### Assessment of tic severity

Tic severity was assessed using the YGTSS. The global severity score was calculated as the sum of the total tic score (combined motor and vocal tic domains; possible range, 0–50) and the impairment score (possible range, 0–50), yielding a possible range of 0–100. The source data confirmed this calculation for all 41 participants. The total tic score and impairment score were available for exploratory component analyses; however, individual frequency and intensity item scores were not retained in a sufficiently complete and standardized form for reliable analysis.

### Laboratory variables

Serum vitamin variables included 25(OH)D, 25(OH)D2, 25(OH)D3, vitamin A, vitamin E, and vitamin K. Immune variables included lymphocyte absolute count; T-, B-, and NK-cell percentages; CD4^+^ and CD8^+^ T-cell percentages; CD4^+^ and CD8^+^ T-cell absolute counts; and the CD4/CD8 ratio. Laboratory results were obtained from the hospital clinical laboratory records. For subgroup analysis, vitamin D status was categorized as <20, 20 to <30, and ≥30 ng/mL according to the 2011 Endocrine Society criteria used in the original study protocol (23).

### Statistical analysis

Continuous variables are presented as mean ± standard deviation, median with interquartile range, and range. Categorical variables are presented as counts and percentages. Normality was assessed using the Shapiro–Wilk test, with detailed results presented in **Supplementary Table S3**. Spearman rank correlation was used to assess unadjusted monotonic associations between the YGTSS global severity score and vitamin or lymphocyte subset variables without requiring normally distributed variables or a strictly linear relationship. Pearson correlations were retained as supplementary analyses. The 15 Spearman correlations constituted the primary multiplicity-controlled association analyses, and multiple testing was controlled using the Benjamini–Hochberg false discovery rate method. Separate adjusted regression models, YGTSS component analyses, and vitamin D subgroup comparisons were treated as secondary exploratory analyses. *P*-values from the separate regression models were nominal and were interpreted in the context of the primary FDR-corrected findings.

Differences in the YGTSS global severity score among vitamin D status groups were assessed using the Kruskal–Wallis test. Significant omnibus results were followed by Dunn’s pairwise tests with Bonferroni correction. Linear regression was used to estimate covariate-adjusted associations between each laboratory variable and the mean YGTSS global severity score. Each laboratory variable was entered into a separate model adjusted for age, sex, and disease duration to limit overfitting. Regression diagnostics showed non-normal residuals and several influential observations. HC3 heteroscedasticity-consistent robust standard errors and confidence intervals were used to reduce the sensitivity of inference to variance misspecification and leverage in this small sample; HC3 does not alter the regression coefficient or correct non-linear model specification. A sensitivity analysis additionally adjusted the CD4^+^ T-cell model for total lymphocyte count. Statistical analyses were performed using IBM SPSS Statistics version 27.0 and R version 4.4.2. All tests were two-sided, and *P* < 0.05 was considered statistically significant.

## Results

### Participant characteristics

The study included 41 children: 29 male participants (70.7%) and 12 female participants (29.3%). The mean age was 8.61 ± 2.70 years, and the median disease duration was 1.00 months (IQR, 0.50–6.00). The mean YGTSS global severity score was 30.71 ± 10.25; its total tic and impairment components had means of 15.34 ± 4.17 and 15.37 ± 7.45, respectively. The mean serum 25(OH)D concentration was 20.90 ± 9.15 ng/mL, and the mean CD4^+^ T-cell absolute count was 823.56 ± 243.63 cells/µL (**Table 1**).

**Table 1:** Descriptive characteristics of the study population.

Variable	*n*	Mean ± SD	Median (Q1, Q3)	Range
Age (years)	41	8.61 ± 2.70	8.50 (6.20, 10.70)	4.30–13.40
Disease duration (months)	41	4.17 ± 5.87	1.00 (0.50, 6.00)	0.25–24.00
YGTSS global severity score	41	30.71 ± 10.25	34.00 (22.00, 38.00)	11.00–50.00
YGTSS total tic score	41	15.34 ± 4.17	15.00 (12.00, 18.00)	7.00–26.00
YGTSS impairment score	41	15.37 ± 7.45	20.00 (10.00, 20.00)	0.00–30.00
25(OH)D (ng/mL)	41	20.90 ± 9.15	19.27 (14.18, 27.12)	3.25–42.29
25(OH)D2 (ng/mL)	41	0.85 ± 1.17	0.50 (0.25, 1.06)	0.00–6.84
25(OH)D3 (ng/mL)	41	20.05 ± 8.74	18.81 (13.34, 25.81)	2.52–40.97
Vitamin A (ng/mL)	41	396.86 ± 153.83	354.19 (294.81, 477.37)	166.65–885.04
Vitamin E (µg/mL)	41	8.55 ± 3.58	7.13 (6.27, 9.50)	5.18–19.39
Vitamin K (ng/mL)	41	0.52 ± 0.59	0.27 (0.14, 0.85)	0.01–3.18
Lymphocyte count (cells/µL)	41	2,389.61 ± 612.69	2,417.00 (1,854.00, 2,758.00)	1,334.00–3,980.00
T lymphocytes (%)	41	68.40 ± 6.81	68.88 (62.79, 74.12)	52.56–80.87
B lymphocytes (%)	41	15.84 ± 4.14	15.73 (12.96, 18.43)	9.70–31.41
NK cells (%)	41	14.56 ± 6.51	15.32 (10.19, 18.39)	2.43–35.29
CD4^+^ T cells (%)	41	34.49 ± 5.13	34.86 (30.42, 38.47)	22.26–45.08
CD8^+^ T cells (%)	41	26.52 ± 7.08	26.80 (20.06, 33.01)	13.05–40.75
CD4^+^ T-cell count (cells/µL)	41	823.56 ± 243.63	833.14 (623.19, 1,013.52)	491.91–1,426.33
CD8^+^ T-cell count (cells/µL)	41	629.21 ± 224.61	623.54 (470.37, 756.04)	276.40–1,236.88
CD4/CD8 ratio	41	1.41 ± 0.48	1.17 (1.09, 1.63)	0.55–3.01

Data are shown as mean ± SD, median (Q1, Q3), and range. Cell-subset percentages are expressed as percentage points.

YGTSS, Yale Global Tic Severity Scale; 25(OH)D, 25-hydroxyvitamin D.

### Correlations between YGTSS global severity score and laboratory variables

In Spearman correlation analyses, CD4^+^ T-cell absolute count showed the strongest inverse association with the YGTSS global severity score (*ρ* = −0.474, *P* = 0.0017). This association remained statistically significant after FDR correction (adjusted *P* = 0.0260). Lymphocyte count (*ρ* = −0.359, *P* = 0.0212), 25(OH)D3 (*ρ* = −0.352, *P* = 0.0240), and total 25(OH)D (*ρ* = −0.331, *P* = 0.0344) were nominally associated with the global severity score but did not remain significant after FDR correction (**Table 2**). The corresponding scatterplots are shown in **Figures 1**–**4**, and the ranked correlations for all laboratory variables are summarized in (**Figure 5**).

**Figure 1:**
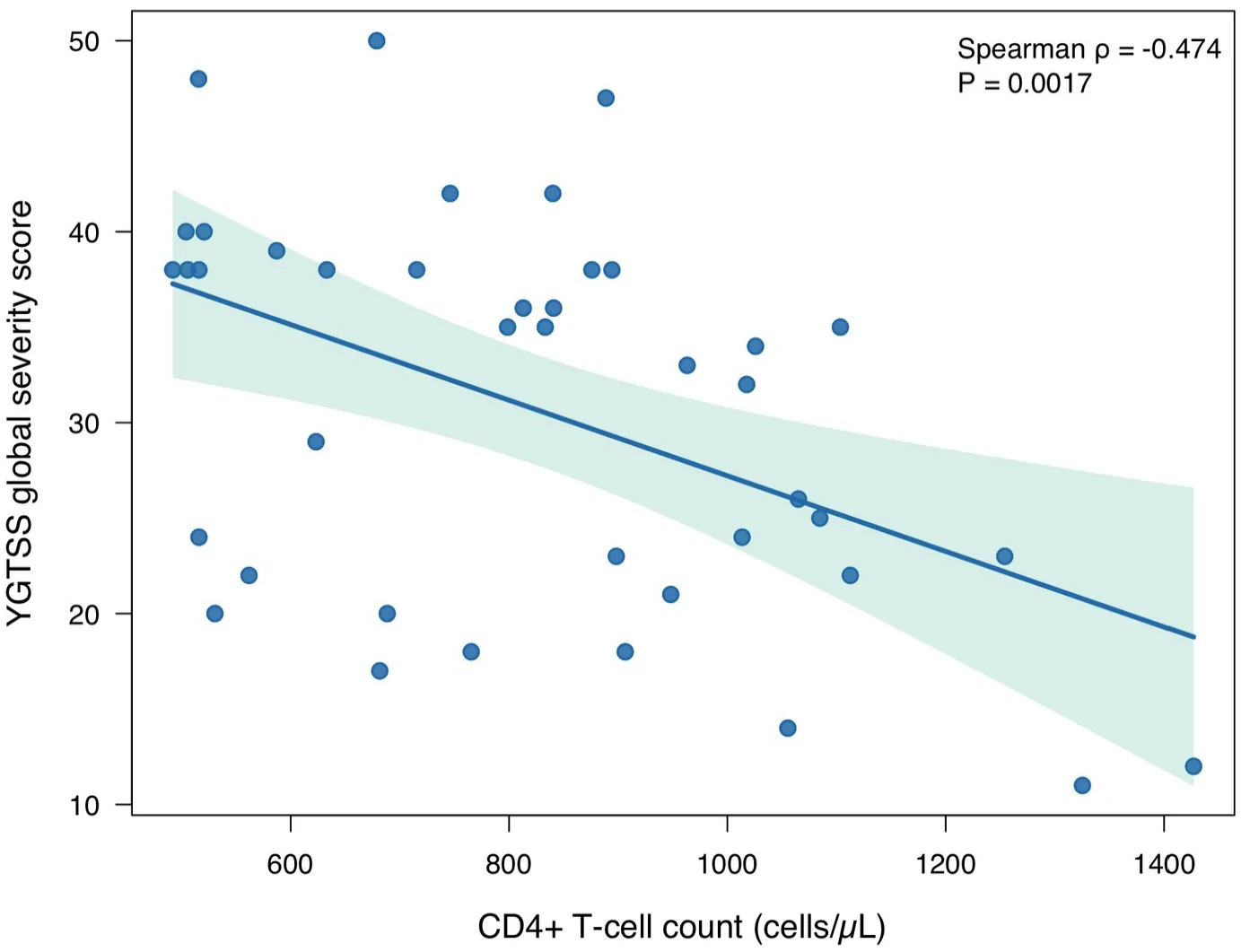
Scatter plot showing the inverse association between the YGTSS global severity score and CD4^+^ T-cell absolute count. The fitted line and 95% confidence band are shown for visualization only.

**Figure 2:**
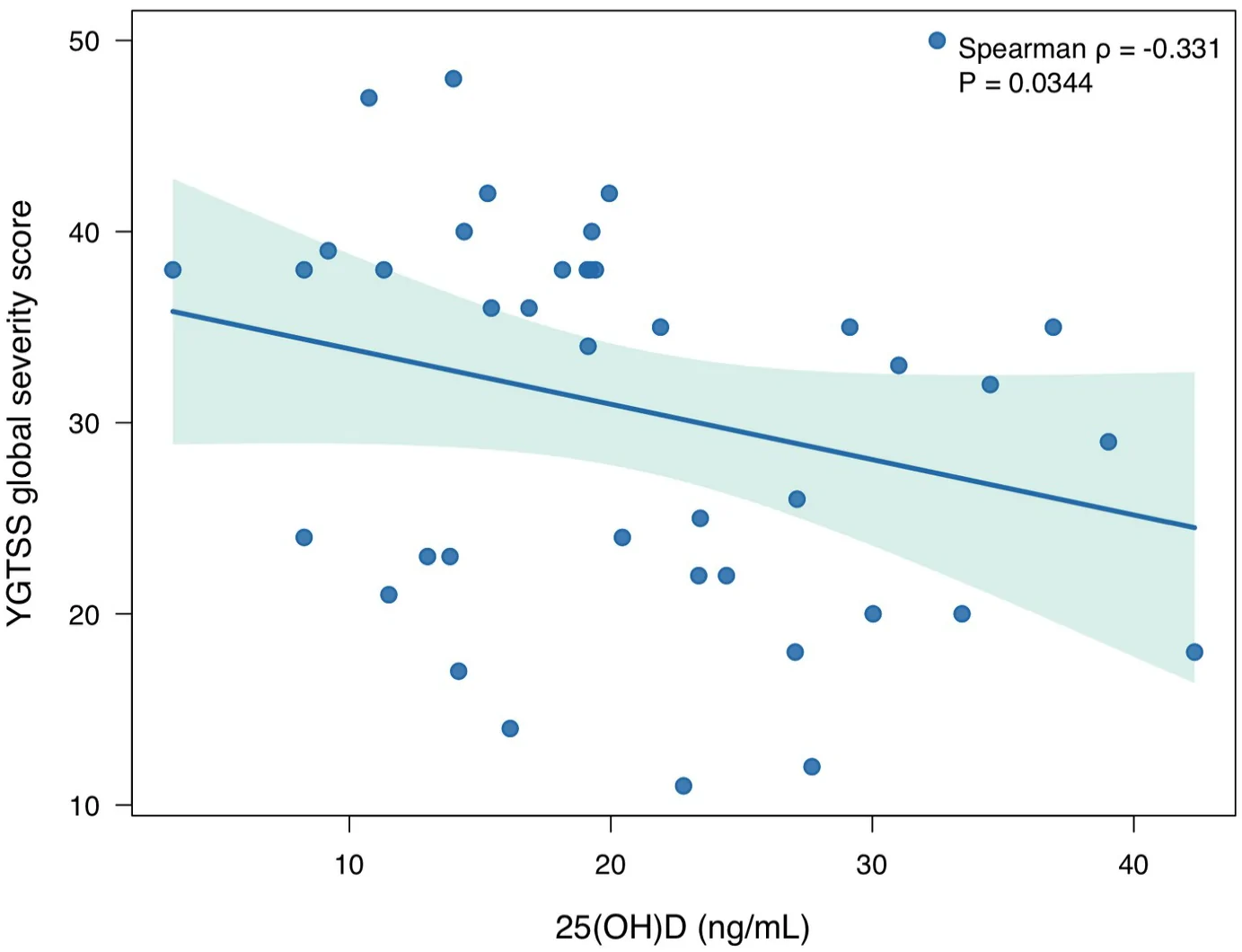
Scatter plot showing the relationship between the YGTSS global severity score and serum 25(OH)D. The fitted line and 95% confidence band are shown for visualization only.

**Figure 3:**
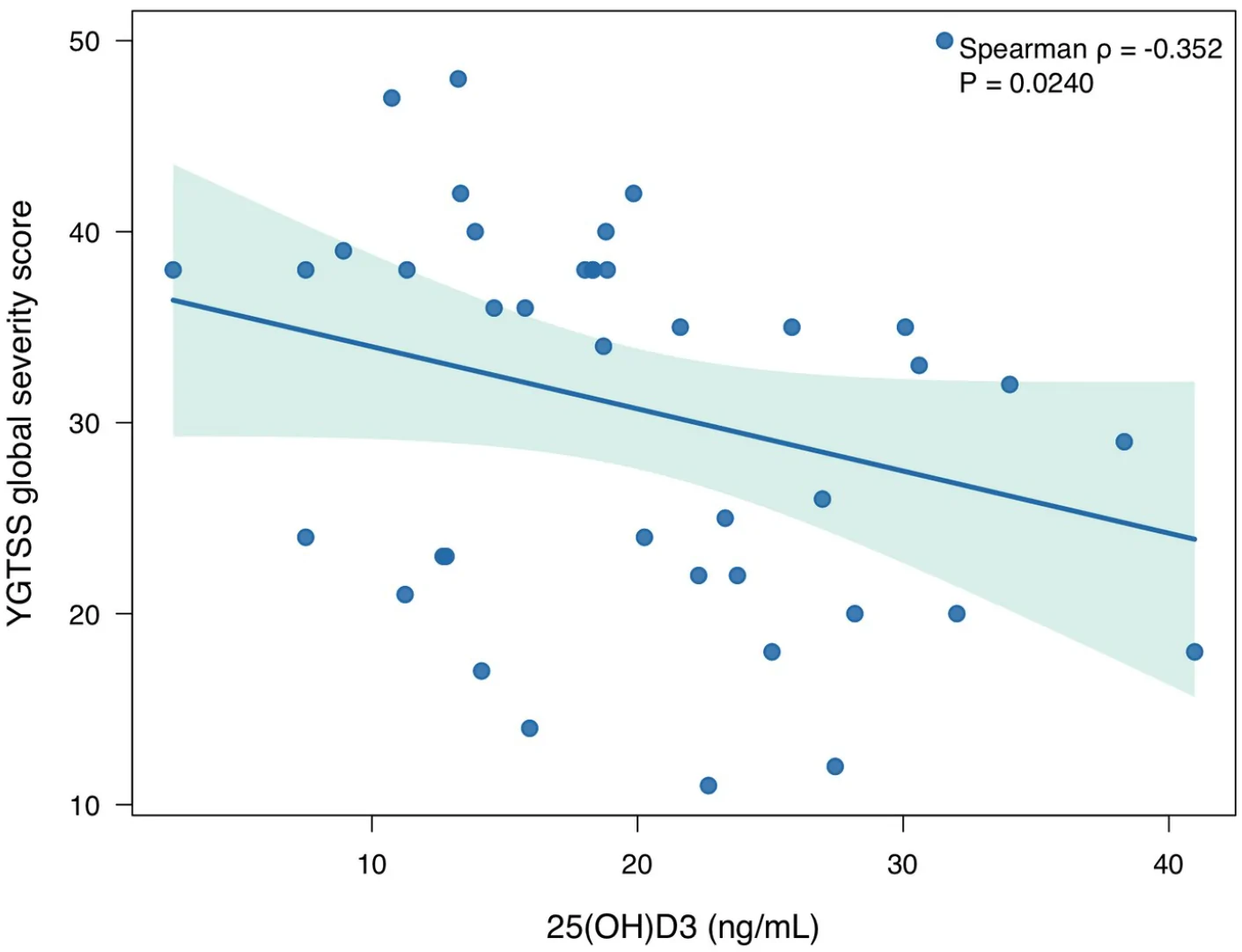
Scatter plot showing the relationship between the YGTSS global severity score and serum 25(OH)D3. The fitted line and 95% confidence band are shown for visualization only.

**Figure 4:**
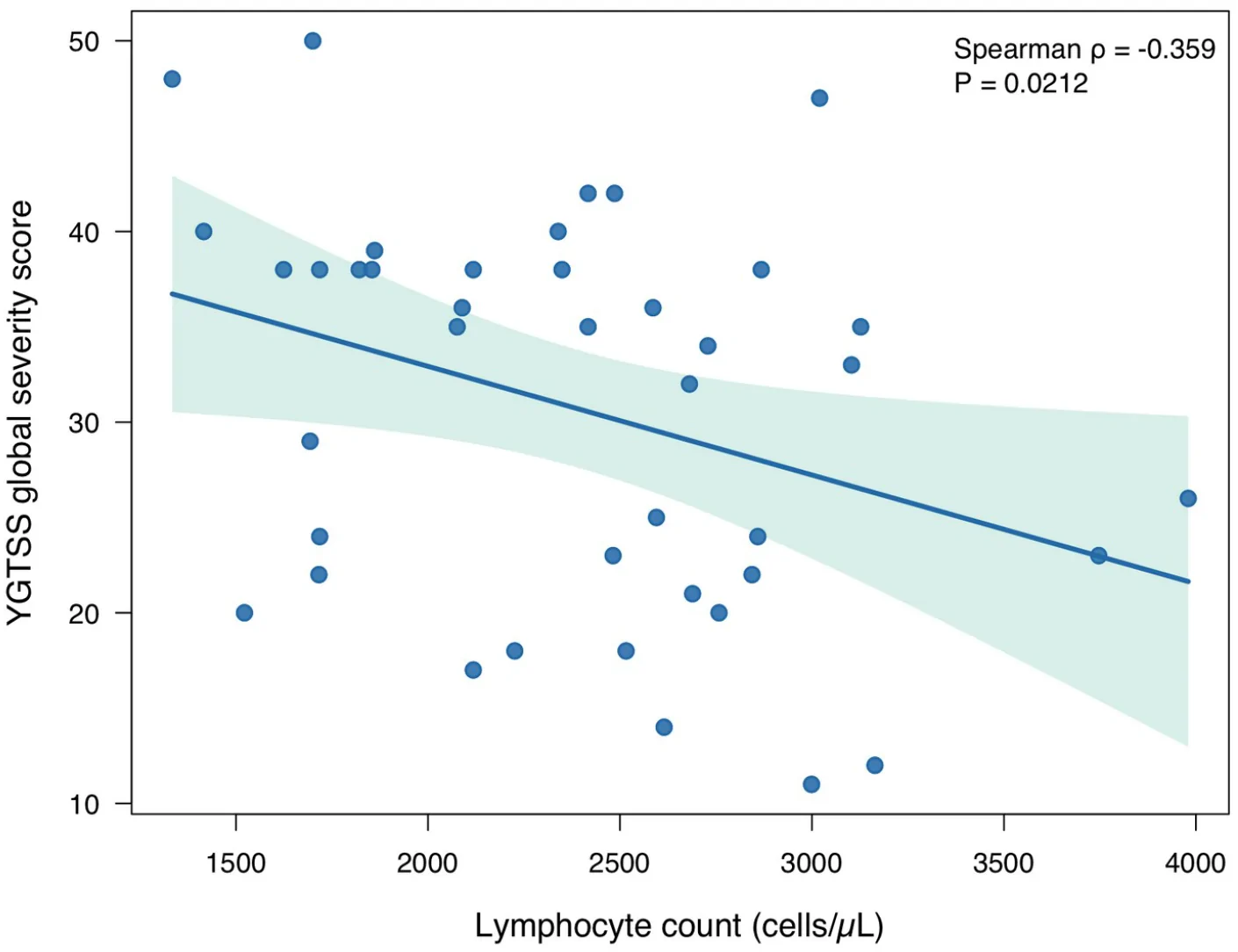
Scatter plot showing the relationship between the YGTSS global severity score and lymphocyte absolute count. The fitted line and 95% confidence band are shown for visualization only.

**Table 2:** Spearman correlations between the YGTSS global severity score and laboratory variables.

Laboratory variable	*n*	Spearman *ρ*	*P*-value	FDR-adjusted *P*
CD4^+^ T-cell count (cells/µL)	41	−0.474	0.0017	0.0260
Lymphocyte count (cells/µL)	41	−0.359	0.0212	0.1201
25(OH)D3 (ng/mL)	41	−0.352	0.0240	0.1201
25(OH)D (ng/mL)	41	−0.331	0.0344	0.1289
CD4^+^ T cells (%)	41	−0.280	0.0761	0.2284
CD8^+^ T-cell count (cells/µL)	41	−0.184	0.2482	0.6205
NK cells (%)	41	−0.125	0.4349	0.9319
Vitamin K (ng/mL)	41	−0.074	0.6467	0.9872
Vitamin A (ng/mL)	41	0.069	0.6702	0.9872
Vitamin E (µg/mL)	41	−0.063	0.6950	0.9872
CD8^+^ T cells (%)	41	0.021	0.8945	0.9872
B lymphocytes (%)	41	−0.019	0.9040	0.9872
CD4/CD8 ratio	41	0.006	0.9697	0.9872
25(OH)D2 (ng/mL)	41	0.005	0.9762	0.9872
T lymphocytes (%)	41	−0.003	0.9872	0.9872

Spearman *ρ* was the primary coefficient. Benjamini–Hochberg FDR correction was applied across 15 laboratory variables. Pearson correlations are shown in **Supplementary Table S1**.

**Figure 5:**
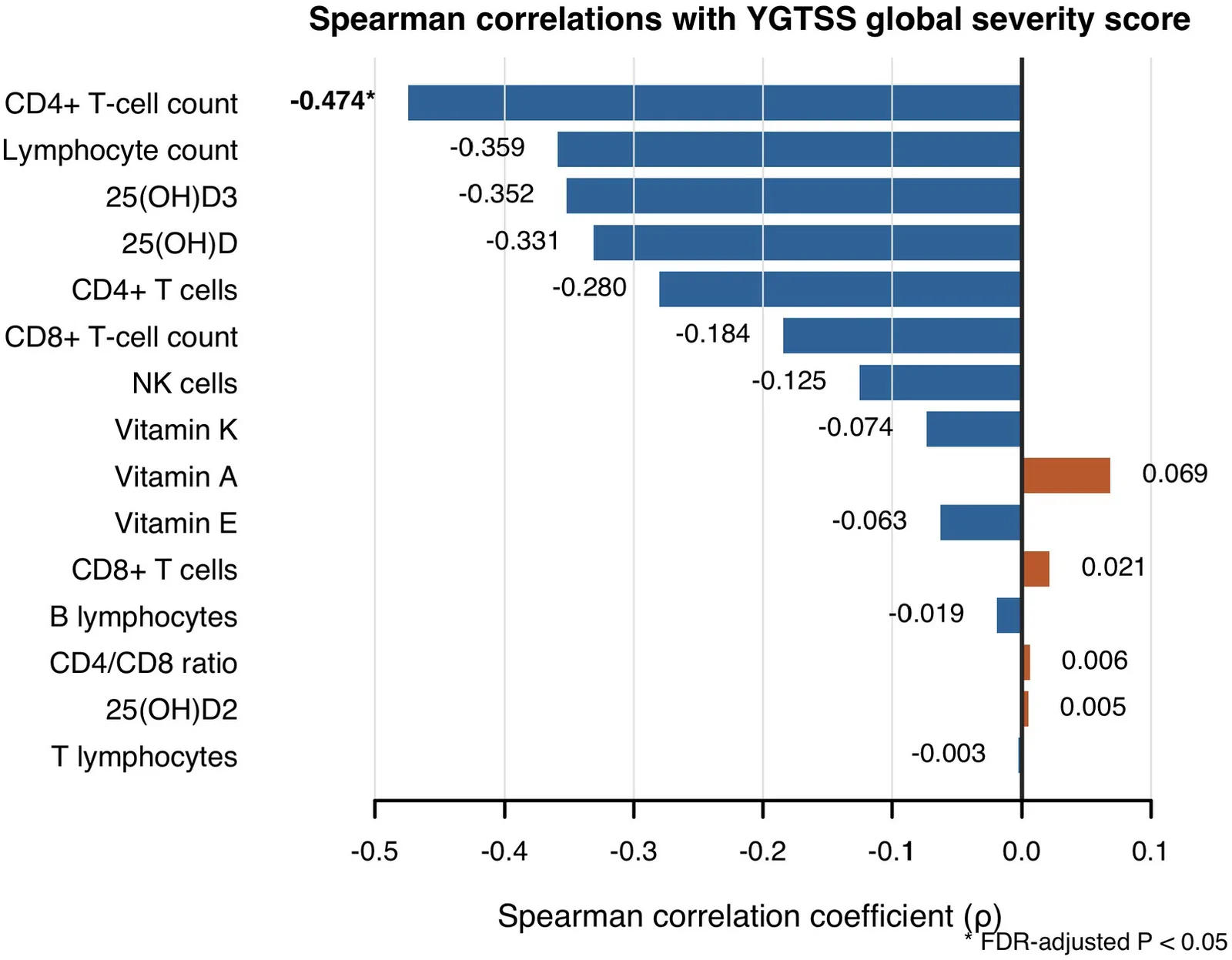
Ranked Spearman correlations between the YGTSS global severity score and vitamin or lymphocyte subset variables. Bars extending to the left indicate inverse correlations, and bars extending to the right indicate positive correlations. The asterisk denotes statistical significance after Benjamini–Hochberg FDR correction (adjusted *P* < 0.05).

### Adjusted regression analyses

After adjustment for age, sex, and disease duration, CD4^+^ T-cell absolute count remained inversely associated with the YGTSS global severity score using HC3 robust standard errors (*B* = −0.018, 95% CI −0.031 to −0.005; *P* = 0.0069). Expressed on a more clinically interpretable scale, every 100 cells/µL higher CD4^+^ T-cell count was associated with an approximately 1.8-point lower YGTSS global severity score (95% CI −3.07 to −0.53; *P* = 0.0069), assuming a linear association. This estimate describes a cross-sectional association and should not be interpreted as the expected effect of increasing CD4^+^ T-cell count. No other laboratory variable had a nominal *P* < 0.05 in the separate adjusted models (**Table 3**). In the sensitivity analysis, the inverse CD4^+^ association persisted after additional adjustment for total lymphocyte count, but the estimate was less precise and close to the nominal significance threshold (*B* = −0.023, 95% CI −0.046 to −0.0004; *P* = 0.0463; **Supplementary Table S5**).

**Table 3:** Adjusted linear regression analyses for the YGTSS global severity score.

Laboratory variable	*n*	*B*	HC3 robust SE	Lower 95% CI	Upper 95% CI	*P*-value
CD4^+^ T-cell count (cells/µL)	41	−0.018	0.006	−0.031	−0.005	0.0069
Lymphocyte count (cells/µL)	41	−0.005	0.003	−0.010	0.000	0.0606
25(OH)D2 (ng/mL)	41	1.386	1.030	−0.704	3.476	0.1869
CD4^+^ T cells (%)	41	−0.452	0.365	−1.192	0.287	0.2231
Vitamin A (ng/mL)	41	0.009	0.008	−0.008	0.025	0.2810
CD8^+^ T-cell count (cells/µL)	41	−0.007	0.006	−0.020	0.006	0.2993
Vitamin K (ng/mL)	41	−1.968	1.900	−5.822	1.887	0.3074
CD4/CD8 ratio	41	−4.281	4.831	−14.079	5.518	0.3815
NK cells (%)	41	−0.286	0.344	−0.983	0.411	0.4115
25(OH)D3 (ng/mL)	41	−0.203	0.262	−0.734	0.328	0.4435
25(OH)D (ng/mL)	41	−0.153	0.251	−0.662	0.355	0.5445
CD8^+^ T cells (%)	41	0.123	0.255	−0.394	0.640	0.6315
Vitamin E (µg/mL)	41	0.227	0.503	−0.792	1.247	0.6539
B lymphocytes (%)	41	0.123	0.486	−0.863	1.108	0.8020
T lymphocytes (%)	41	0.040	0.251	−0.470	0.550	0.8743

Each laboratory variable was entered into a separate model adjusted for age, sex, and disease duration. HC3 robust standard errors and confidence intervals are reported. Coefficients for cell percentages represent a one-percentage-point increase. *P*-values were nominal and were not corrected for multiple testing.

*B*, unstandardized coefficient; CI, confidence interval.

Exploratory analyses of the available YGTSS components showed inverse associations of CD4^+^ T-cell count with the total tic score (*ρ* = −0.412, *P* = 0.0075) and impairment score (*ρ* = −0.412, *P* = 0.0074). Neither component-level association remained significant after FDR correction (adjusted *P* = 0.1127 and 0.1114, respectively; **Supplementary Table S2**). Item-level frequency and intensity scores were unavailable for reliable analysis.

### YGTSS global severity score according to vitamin D status

When participants were grouped by serum 25(OH)D status, 23 children had <20 ng/mL, 10 had 20 to <30 ng/mL, and 8 had ≥30 ng/mL. The <20-ng/mL group had the highest mean YGTSS global severity score (34.43 ± 9.31). The omnibus difference was significant (Kruskal–Wallis *H* = 9.848, *P* = 0.0073). Dunn–Bonferroni comparisons showed a significant difference between the <20- and 20- to <30-ng/mL groups (adjusted *P* = 0.0077), but not between the <20- and ≥30-ng/mL groups (adjusted *P* = 0.2775) or between the 20- to <30- and ≥30-ng/mL groups (adjusted *P* = 1.0000; **Table 4**; **Figure 6**; **Supplementary Table S4**). These exploratory pairwise findings did not show a monotonic dose–response pattern, and their stability was limited by the small and unequal group sizes.

**Table 4:** YGTSS global severity score according to serum 25(OH)D status.

25(OH)D group	*n*	YGTSS global severity score, mean ± SD	YGTSS global severity score, median (Q1, Q3)
<20 ng/mL	23	34.43 ± 9.31	38.00 (29.00, 39.50)
20 to <30 ng/mL	10	23.00 ± 8.12	23.00 (19.00, 25.75)
≥30 ng/mL	8	29.62 ± 10.57	30.50 (20.00, 33.50)

Kruskal–Wallis *H* = 9.848, *P* = 0.0073. Dunn’s pairwise tests with Bonferroni correction: <20 vs. 20 to <30 ng/mL, adjusted *P* = 0.0077; <20 vs. ≥30 ng/mL, adjusted *P* = 0.2775; 20 to <30 vs. ≥30 ng/mL, adjusted *P* = 1.0000.

**Figure 6:**
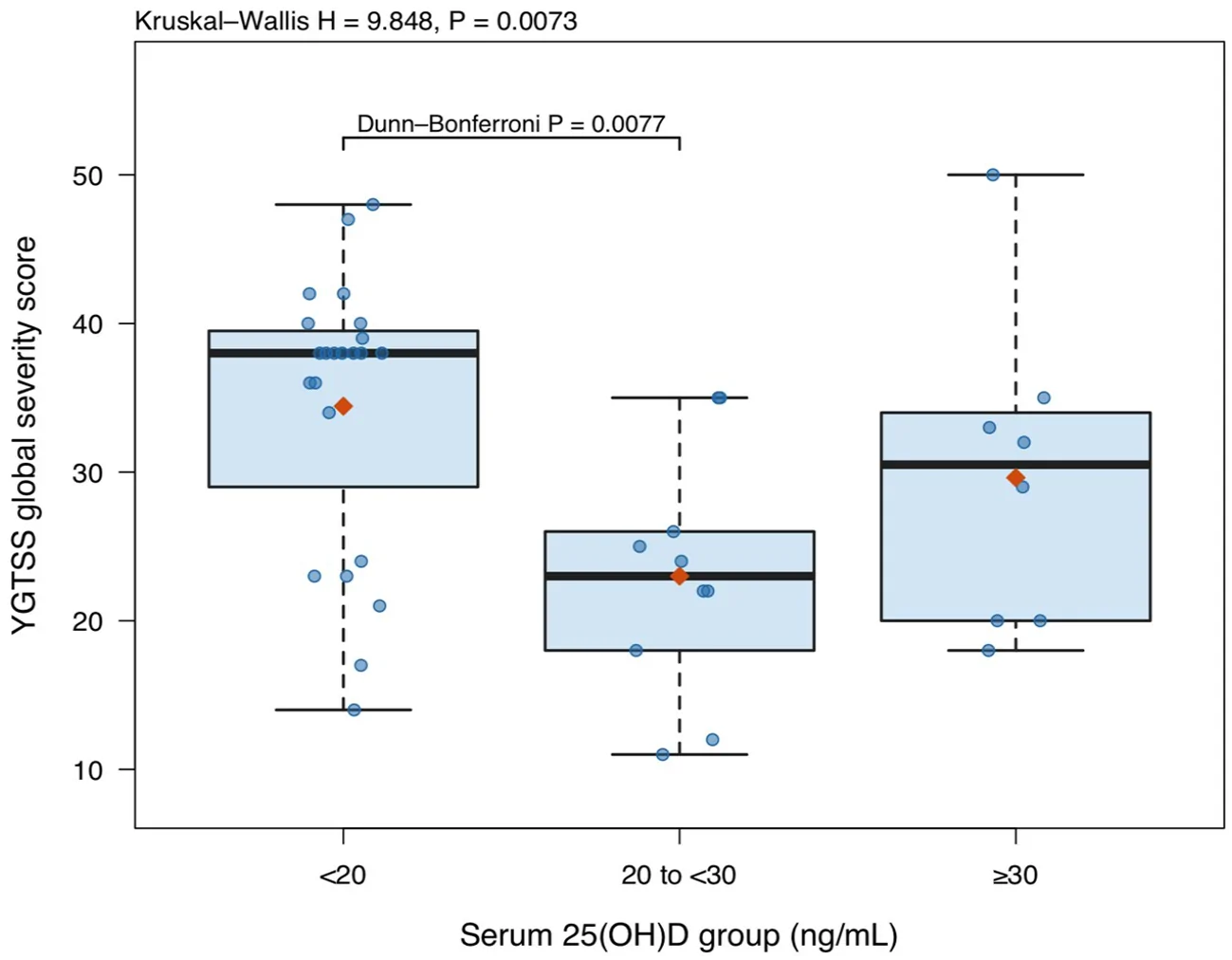
Distribution of the YGTSS global severity score by serum 25(OH)D status. Circles represent individual observations, and orange diamonds represent group means. The bracket identifies the significant Dunn–Bonferroni pairwise comparison.

## Discussion

In this exploratory cross-sectional study of children with tic disorders, lower peripheral CD4^+^ T-cell absolute count was associated with a higher YGTSS global severity score, and the association persisted after demographic and disease-duration adjustment, use of HC3 robust standard errors, and additional adjustment for total lymphocyte count. Vitamin D associations were less robust. Although the vitamin D-deficient group had the highest severity scores, only one pairwise contrast was significant, and the categories did not show a monotonic dose–response pattern.

Because the YGTSS global severity score combines the total tic score and impairment score, the primary finding should be interpreted as an association with overall tic-related symptom burden rather than with tic frequency, tic intensity, or functional impairment alone. Exploratory component analyses showed nominal inverse associations of CD4^+^ T-cell count with both the total tic score and impairment score, but neither component-level association remained significant after FDR correction. The main association therefore cannot be attributed specifically to tic manifestations or functional impairment.

The most notable finding was the adjusted inverse association between CD4^+^ T-cell absolute count and tic-related symptom burden measured by the YGTSS global severity score. Previous studies have suggested that tic disorders may involve immune dysregulation, including altered cytokine expression and lymphocyte subset imbalance (8–14). In children with tic disorders and allergic diseases, changes in CD3^+^ T cells, CD4^+^ T cells, and the CD4/CD8 ratio have been reported and may relate to clinical severity (14). Our findings extend this literature by showing that the CD4^+^ T-cell association persisted after adjustment for demographic and disease-duration covariates and in a sensitivity model that included total lymphocyte count.

CD4^+^ T-cell absolute count and percentage capture different aspects of peripheral immune status. The absolute count reflects the number of circulating CD4^+^ cells per unit of blood, whereas the percentage reflects their relative distribution among lymphocytes. The association observed for the absolute count, but not the percentage, may therefore partly reflect broader variation in circulating lymphocyte levels rather than a CD4-specific proportional change. Although the association persisted after additional adjustment for total lymphocyte count, the sensitivity estimate was imprecise and close to the nominal significance threshold. The effect estimate per 100 cells/µL should likewise be interpreted as a cross-sectional association rather than the effect of an intervention.

The biological interpretation of reduced CD4^+^ T-cell count in relation to higher tic severity remains uncertain. CD4^+^ T cells include heterogeneous functional subsets, including Th1, Th2, Th17, and regulatory T-cell populations, which may exert divergent effects on neuroinflammation, immune tolerance, and cytokine networks. Prior reviews of Tourette syndrome have emphasized possible immune-mediated mechanisms, including post-infectious immune activation, inflammatory cytokines, and impaired immune regulation (8, 9). A meta-analysis also reported altered T-cell profiles in pediatric Tourette syndrome, including reduced CD4^+^ T cells and increased proinflammatory cytokines such as IL-6 and TNF-alpha (13). Because our dataset did not include functional T-cell phenotyping, regulatory T cells, Th17 cells, or cytokine panels, the observed association should be interpreted as a peripheral immune signal rather than evidence of a specific causal pathway.

Several mechanisms may explain the observed inverse relationship between CD4^+^ T-cell absolute count and tic severity. First, reduced circulating CD4^+^ T-cell count may reflect immune redistribution or persistent immune activation, whereby activated T cells migrate from peripheral blood to inflammatory sites or lymphoid tissues. Second, a lower CD4^+^ T-cell count may indicate impaired immune regulation, particularly if regulatory T-cell function is reduced. Third, CD4^+^ T-cell alterations may interact with cytokine-mediated neuroinflammatory pathways that influence dopaminergic neurotransmission, microglial activation, and cortico-striato-thalamo-cortical circuit function, all of which are relevant to tic generation and severity (8, 9, 13).

The vitamin D findings require cautious interpretation. Total 25(OH)D and 25(OH)D3 were nominally inversely correlated with the YGTSS global severity score, but neither association survived FDR correction or covariate adjustment. The significant Dunn–Bonferroni contrast was limited to the <20- vs. 20- to <30-ng/mL groups; the ≥30-ng/mL group did not differ significantly from either group. Thus, these data do not demonstrate a monotonic dose–response relationship. The ≥30-ng/mL group included only eight participants, and the small and unequal group sizes may have produced unstable estimates. The findings are directionally compatible with earlier observational studies (18, 19) and with the immunomodulatory effects of vitamin D (15–17), but residual confounding and chance remain plausible explanations.

This study has several strengths. It included newly diagnosed, treatment-naive children, thereby reducing confounding by tic-directed medications. It also jointly examined serum vitamin indices and lymphocyte subsets in relation to a standardized tic severity scale and applied multiple complementary analyses, including Spearman correlation, FDR correction, vitamin D subgroup comparison, and adjusted regression.

Several limitations should be acknowledged. First, the single-center sample was small, limiting power, precision, and generalizability. Second, the cross-sectional design precludes causal inference. Third, no healthy control group was included; the findings therefore concern severity-related associations within children with tic disorders and cannot establish whether vitamin or immune measures differ from healthy children. Fourth, body mass index, season of blood collection, sunlight exposure, dietary intake, allergic disease, and detailed psychiatric comorbidity data were unavailable. Fifth, infection was excluded clinically, but high-sensitivity C-reactive protein was available for only 24 of 41 participants and was not a standardized eligibility screen; subtle or subclinical inflammation could not be excluded. Sixth, lymphocyte subset values in children vary with age. Analyses were adjusted for age, but age-standardized *z* scores and local age-specific reference intervals were unavailable; therefore, the findings do not establish that CD4^+^ T-cell counts were abnormally low compared with those of age-matched healthy children. Seventh, individual YGTSS frequency and intensity items were not consistently retained; analyses of the available total tic and impairment components were exploratory and did not survive FDR correction. Eighth, although the CD4^+^ association persisted after additional adjustment for total lymphocyte count, the estimate was imprecise and close to the significance threshold. Finally, residual non-normality and several influential observations were present; HC3 robust standard errors were therefore used, but larger prospective studies remain necessary.

The present findings have no immediate role in diagnosing tic disorders, classifying patients, predicting an individual clinical course, or selecting treatment. They do not support using vitamin D concentration or CD4^+^ T-cell count alone for these purposes. Current guidelines emphasize clinical assessment, functional impairment, comorbidities, and individualized management rather than biomarker-based classification (6, 7). Evaluation of vitamin D status may still be appropriate when a child has established clinical risk factors for deficiency, independent of its proposed role as a tic-related biomarker. The observed CD4^+^ T-cell association should be regarded as a hypothesis-generating signal that requires validation in larger longitudinal and controlled studies.

## Conclusion

Among newly diagnosed, treatment-naive children with tic disorders, lower peripheral CD4^+^ T-cell absolute count was associated with a higher YGTSS global severity score. This association remained significant in the primary adjusted model and persisted at the nominal significance threshold in a lymphocyte-adjusted sensitivity analysis, although the latter estimate was imprecise. Vitamin D findings were exploratory and did not support a clear dose–response relationship. Prospective studies with larger samples, healthy controls, age-standardized immune measures, and detailed YGTSS item data are warranted.

## Data Availability

The individual-level data are not publicly available due to participant privacy and institutional restrictions. Requests for access may be directed to the corresponding author, Jian Zhang (lucky1975@163.com), and will be considered subject to institutional approval and applicable ethical requirements.
