# Supplementary material for: Associations of vitamin status and T-lymphocyte subsets with tic severity in children: a cross-sectional study using the Yale Global Tic Severity Scale
Source: Front Psychiatry. 2026 Oct 14;17:1903047. doi: 10.3389/fpsyt.2026.1903047 (PMC13713763; doi:10.3389/fpsyt.2026.1903047)
Supplement: Supplementary file 1 [file SupplementaryFile1.docx]

**Supplementary Material**

**Supplementary Table S1. Pearson correlations with the YGTSS global severity score**

| **Laboratory variable** | **n** | **Pearson r** | **P value** |
| --- | --- | --- | --- |
| CD4+ T-cell count (cells/µL) | 41 | -0.470 | 0.0019 |
| Lymphocyte count (cells/µL) | 41 | -0.341 | 0.0293 |
| 25(OH)D3 (ng/mL) | 41 | -0.278 | 0.0789 |
| 25(OH)D (ng/mL) | 41 | -0.258 | 0.1027 |
| CD4+ T cells (%) | 41 | -0.269 | 0.0890 |
| CD8+ T-cell count (cells/µL) | 41 | -0.211 | 0.1853 |
| NK cells (%) | 41 | -0.122 | 0.4473 |
| Vitamin K (ng/mL) | 41 | -0.155 | 0.3336 |
| Vitamin A (ng/mL) | 41 | 0.077 | 0.6327 |
| Vitamin E (µg/mL) | 41 | 0.038 | 0.8140 |
| CD8+ T cells (%) | 41 | 0.048 | 0.7657 |
| B lymphocytes (%) | 41 | -0.041 | 0.8009 |
| CD4/CD8 ratio | 41 | -0.182 | 0.2547 |
| 25(OH)D2 (ng/mL) | 41 | 0.053 | 0.7403 |
| T lymphocytes (%) | 41 | -0.003 | 0.9830 |

Pearson correlations are supplementary; Spearman correlations were the primary analyses.

**Supplementary Table S2. Exploratory correlations with available YGTSS components**

| **Laboratory variable** | **Total tic ρ** | **P** | **FDR P** | **Impairment ρ** | **P** | **FDR P** |
| --- | --- | --- | --- | --- | --- | --- |
| CD4+ T-cell count (cells/µL) | -0.412 | 0.0075 | 0.1126 | -0.412 | 0.0074 | 0.1114 |
| Lymphocyte count (cells/µL) | -0.376 | 0.0154 | 0.1156 | -0.277 | 0.0799 | 0.3996 |
| 25(OH)D3 (ng/mL) | -0.330 | 0.0350 | 0.1750 | -0.218 | 0.1715 | 0.5146 |
| 25(OH)D (ng/mL) | -0.312 | 0.0470 | 0.1761 | -0.198 | 0.2152 | 0.5380 |
| CD4+ T cells (%) | -0.153 | 0.3411 | 0.7888 | -0.283 | 0.0731 | 0.3996 |
| CD8+ T-cell count (cells/µL) | -0.144 | 0.3681 | 0.7888 | -0.244 | 0.1240 | 0.4651 |
| NK cells (%) | -0.194 | 0.2245 | 0.6736 | -0.052 | 0.7451 | 0.7566 |
| Vitamin K (ng/mL) | -0.037 | 0.8167 | 0.9424 | -0.117 | 0.4658 | 0.7036 |
| Vitamin A (ng/mL) | 0.085 | 0.5985 | 0.8161 | 0.087 | 0.5903 | 0.7036 |
| Vitamin E (µg/mL) | -0.003 | 0.9848 | 0.9848 | -0.093 | 0.5628 | 0.7036 |
| CD8+ T cells (%) | 0.085 | 0.5951 | 0.8161 | -0.109 | 0.4960 | 0.7036 |
| B lymphocytes (%) | -0.090 | 0.5740 | 0.8161 | 0.050 | 0.7566 | 0.7566 |
| CD4/CD8 ratio | -0.010 | 0.9530 | 0.9848 | 0.127 | 0.4277 | 0.7036 |
| 25(OH)D2 (ng/mL) | -0.049 | 0.7631 | 0.9424 | 0.082 | 0.6098 | 0.7036 |
| T lymphocytes (%) | 0.125 | 0.4358 | 0.8161 | -0.118 | 0.4612 | 0.7036 |

The total tic score is the combined motor and vocal tic-domain score. Individual YGTSS frequency and intensity items were unavailable for reliable analysis. FDR correction was applied separately within each component analysis.

**Supplementary Table S3. Shapiro–Wilk tests of normality**

| **Variable** | **n** | **W** | **P value** | **Normal at α=0.05** |
| --- | --- | --- | --- | --- |
| Age (years) | 41 | 0.951 | 0.0750 | Yes |
| Disease duration (months) | 41 | 0.690 | <0.0001 | No |
| YGTSS global severity score | 41 | 0.952 | 0.0808 | Yes |
| 25(OH)D (ng/mL) | 41 | 0.975 | 0.4924 | Yes |
| 25(OH)D2 (ng/mL) | 41 | 0.603 | <0.0001 | No |
| 25(OH)D3 (ng/mL) | 41 | 0.982 | 0.7326 | Yes |
| Vitamin A (ng/mL) | 41 | 0.911 | 0.0037 | No |
| Vitamin E (µg/mL) | 41 | 0.816 | <0.0001 | No |
| Vitamin K (ng/mL) | 41 | 0.756 | <0.0001 | No |
| Lymphocyte count (cells/µL) | 41 | 0.970 | 0.3353 | Yes |
| T lymphocytes (%) | 41 | 0.972 | 0.3905 | Yes |
| B lymphocytes (%) | 41 | 0.910 | 0.0034 | No |
| NK cells (%) | 41 | 0.952 | 0.0827 | Yes |
| CD4+ T cells (%) | 41 | 0.985 | 0.8594 | Yes |
| CD8+ T cells (%) | 41 | 0.971 | 0.3674 | Yes |
| CD4+ T-cell count (cells/µL) | 41 | 0.950 | 0.0712 | Yes |
| CD8+ T-cell count (cells/µL) | 41 | 0.963 | 0.1961 | Yes |
| CD4/CD8 ratio | 41 | 0.875 | 0.0003 | No |

**Supplementary Table S4. Dunn–Bonferroni post-hoc comparisons among vitamin D groups**

| **Comparison** | **Mean rank 1** | **Mean rank 2** | **Z** | **Unadjusted P** | **Adjusted P** |
| --- | --- | --- | --- | --- | --- |
| <20 ng/mL vs 20 to <30 ng/mL | 25.93 | 12.30 | 3.014 | 0.0026 | 0.0077 |
| <20 ng/mL vs ≥30 ng/mL | 25.93 | 17.69 | 1.682 | 0.0925 | 0.2775 |
| 20 to <30 ng/mL vs ≥30 ng/mL | 12.30 | 17.69 | -0.951 | 0.3416 | 1.0000 |

The omnibus Kruskal–Wallis test yielded H = 9.848, df = 2 and P = 0.0073. Adjusted P values use Bonferroni correction for three pairwise comparisons.

**Supplementary Table S5. Sensitivity analyses of the CD4+ T-cell association**

| **Analysis** | **B** | **HC3 robust SE** | **95% CI** | **P value** |
| --- | --- | --- | --- | --- |
| Primary model with HC3 robust standard errors | -0.018 | 0.006 | -0.031 to -0.005 | 0.0069 |
| Additional adjustment for total lymphocyte count with HC3 robust standard errors | -0.023 | 0.011 | -0.046 to -0.000 | 0.0463 |

Both models were adjusted for age, sex and disease duration. The second model was additionally adjusted for total lymphocyte count. B represents the change in YGTSS global severity score per 1 cell/µL increase in CD4+ T-cell count.
